# Olfaction regulates peripheral mitophagy and mitochondrial function

**DOI:** 10.1126/sciadv.adn0014

**Published:** 2024-06-21

**Authors:** Julian G. Dishart, Corinne L. Pender, Koning Shen, Hanlin Zhang, Megan Ly, Madison B. Webb, Andrew Dillin

**Affiliations:** ^1^ The Helen Wills Neuroscience Institute, University of California, Berkeley, Berkeley, CA 94720, USA.; ^2^ Department of Molecular and Cellular Biology, University of California, Berkeley, Berkeley, CA 94720, USA.; ^3^ Howard Hughes Medical Institute, University of California, Berkeley, Berkeley, CA 94720, USA.; ^4^ Department of Plant and Microbial Biology, University of California, Berkeley, Berkeley, CA 94720, USA.

## Abstract

The central nervous system coordinates peripheral cellular stress responses, including the unfolded protein response of the mitochondria (UPR^MT^); however, the contexts for which this regulatory capability evolved are unknown. UPR^MT^ is up-regulated upon pathogenic infection and in metabolic flux, and the olfactory nervous system has been shown to regulate pathogen resistance and peripheral metabolic activity. Therefore, we asked whether the olfactory nervous system in *Caenorhabditis elegans* controls the UPR^MT^ cell nonautonomously. We found that silencing a single inhibitory olfactory neuron pair, AWC, led to robust induction of UPR^MT^ and reduction of oxidative phosphorylation dependent on serotonin signaling and *parkin*-mediated mitophagy. Further, AWC ablation confers resistance to the pathogenic bacteria *Pseudomonas aeruginosa* partially dependent on the UPR^MT^ transcription factor *atfs-1* and fully dependent on mitophagy machinery. These data illustrate a role for the olfactory nervous system in regulating whole-organism mitochondrial dynamics, perhaps in preparation for postprandial metabolic stress or pathogenic infection.

## Teaser

Olfaction in *C. elegans* regulates peripheral UPR^MT^ induction, mitophagy, and pathogen resistance through serotonin signaling.

## INTRODUCTION

Coordinating stress responses across tissues is paramount for maintaining cellular homeostasis and longevity in response to environmental insults, and a robust body of literature has established the central nervous system as a master regulator of cellular stress responses across the whole organism. Activating the unfolded protein response of the endoplasmic reticulum (UPR^ER^) (*1*), the heat shock response (*2*), and the mitochondrial unfolded protein response (UPR^MT^) (*3*, *4*) in neurons results in cell nonautonomous induction of their respective stress response in peripheral tissues. This nonautonomous coordination promotes survival in aging and in conditions of cellular stress, leading to the hypothesis that the nervous system evolved the ability to prepare the organism to withstand noxious environmental conditions. More recently, work on the nonautonomous regulation of UPR^MT^ has focused on the requirement of distinct neural circuits and signaling molecules, as well as a broader description of peripheral mitochondrial phenotypes under conditions of neuronal mitochondria dysfunction. For example, induction of mitochondrial stress in neurons by knockout of *fzo-1*/mitofusin drives peripheral UPR^MT^ induction, enhances mitophagy, and increases pathogen resistance dependent on serotoninergic, tyraminergic, and neuropeptidergic signaling (*5*). Despite the insights into the signaling cascades and neuronal players required for peripheral induction of UPR^MT^ by the central nervous system, the evolutionary context and sensory modalities that necessitate this coordination remain incompletely understood.

The olfactory nervous system has been identified as a nonautonomous regulator of metabolic homeostasis and life span. In mice, olfactory exposure to food odors activates hypothalamic neurons, which signal to the liver to activate UPR^ER^ and to initiate lipid synthesis (*6*), while olfactory neuron ablation abates diet-induced obesity and increases mitochondrial respiration in fat tissue by noradrenergic afferent signaling (*7*). A connection between olfaction and metabolic regulation has also been established in the model organism *Caenorhabditis elegans (C. elegans)*, suggesting an evolutionarily conserved role for olfactory circuits in regulating peripheral physiology. Specifically, loss of function in distinct olfactory neurons extends life span by down-regulating insulin signaling (*8*), and silencing a single olfactory neuron pair, AWC, regulates lipid metabolism and enhanced proteostasis through neuroendocrine control (*9*, *10*). These studies indicate that the olfactory nervous system plays a role in assessing environmental nutritional status and signals to the periphery to prepare for food intake.

All organisms must consume nutrients to survive; however, eating poses substantial cellular hazards. In eating, tissues not only are tasked with metabolizing nutrients but also must withstand and clear toxic metabolites, like mitochondria-generated reactive oxygen species (mtROS), and must defend against food-borne pathogens. Pathogenic infection is a formidable cellular stressor, and organisms, including the bacterivore *C. elegans*, induce various genetic programs to counteract pathogen-associated damage. During infection with the pathogenic bacteria *Pseudomonas aeruginosa*, *C. elegans* activate the UPR^MT^ transcription factor *atfs-1* to resolve mitochondrial damage and to induce innate immune response genes (*11*). Loss of *atfs-1* activity and diminished UPR^MT^ induction results in sensitivity to *P. aeruginosa* infection (*11*). In their natural milieu, *C. elegans* are exposed to myriad commensal and pathogenic bacteria and must discern which are helpful or harmful, primarily through the detection of bacterial metabolites. Olfaction is the mode of detection of such volatile metabolites, indicating that olfactory neurons have privileged access to information about potential pathogenic threats in the environment. We hypothesize that olfactory neurons might be top-most coordinators of UPR^MT^ induction and may serve as an early warning system to prepare the periphery for pathogenic infection. Recent work in *C. elegans* has shown that loss of certain sensory neurons, particularly the olfactory neuron AWC, contributes to *P. aeruginosa* resistance; however, whether olfactory regulation of UPR^MT^ is responsible for this resistance has been left unexplored (*12*).

Here, we find that ablation of the inhibitory olfactory neuron pair AWC is sufficient to drive peripheral activation of UPR^MT^, reduce mitochondrial oxidative phosphorylation (OXPHOS) rates, and reduce mitochondrial DNA (mtDNA). These phenotypes are dependent on serotonergic signaling, suggesting that AWC-mediated olfactory circuitry regulates peripheral UPR^MT^ and mtDNA content cell nonautonomously. Moreover, reduction in OXPHOS and mtDNA content downstream of AWC ablation fully depends on the mitophagy machinery *pdr-1* (parkin homolog), and induction of UPR^MT^ partially depends on *pdr-1*. These mitochondrial phenotypes are recapitulated by exposure to the bacterial metabolite 2,3-pentanedione (2,3-pent), an AWC-silencing odorant, suggesting that olfactory regulation of peripheral mitochondrial mass and function may occur in a naturalistic context. *P. aeruginosa* generates 2,3-pent, suggesting that AWC neurons are tuned to detect this pathogen (*13*). Finally, we find that AWC-mediated *P. aeruginosa* infection resistance is partially dependent on the UPR^MT^ transcription factor *atfs-1* and fully dependent on *pdr-1*, indicating that olfactory neurons can prepare the organism for pathogenic insult through regulation of mitochondrial dynamics.

## RESULTS

### Ablation of olfactory neuron AWC induces UPR^MT^, confers ATFS-1–mediated pathogen resistance, reduces OXPHOS, and depletes mtDNA content

**Fig. 1.**
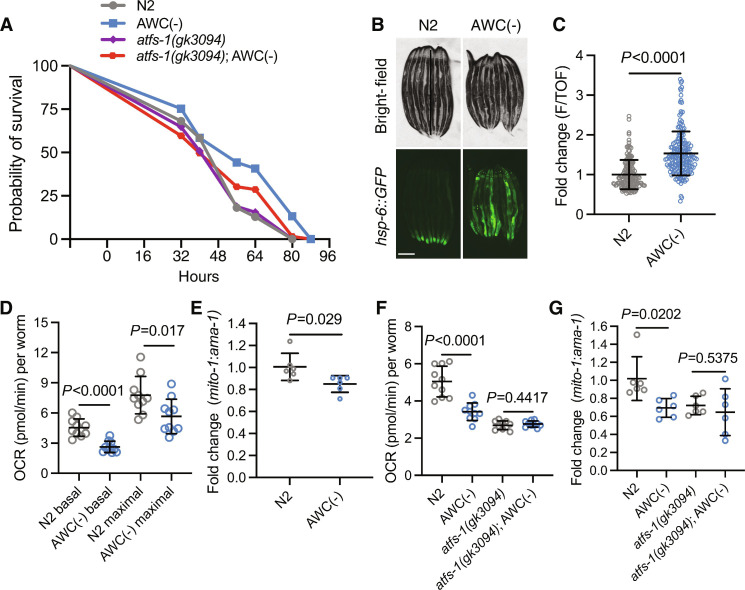
Ablation of olfactory neuron AWC induces UPR^MT^, confers ATFS-1–mediated pathogen resistance, reduces OXPHOS, and depletes mtDNA content. (**A**) Survival of N2, AWC(−), *atfs-1(gk3094)*, and *atfs-1(gk3094)*; AWC(−) on PA14. Log-rank Mantel-Cox test: N2 versus AWC (*P* < 0.0001); *atfs-1(gk3094) versus atfs-1(gk3094)*; AWC(−) (*P* = 0.1201); AWC(−) versus *atfs-1(gk3094)*; AWC(−) (*P* = 0.0027). *N* = 2 biological replicates. (**B**) Representative fluorescent images of *hsp-6::GFP* in N2 and AWC(−) animals. Scale bar, 250 μm. (**C**) Fold change of integrated fluorescence intensity [fluorescence (F)/time of flight (TOF)] measured by bioSorter of *hsp-6::GFP* in strains imaged in (B). Two-tailed unpaired *t* test with Welch’s correction. *N* = 3 biological replicates. (**D**) OCR in N2 without (basal) and with FCCP (maximal). Two-tailed unpaired *t* test with Welch’s correction. *N* = 3 biological replicates. (**E**) Log_2_ fold change of the ratio of *mito-1* to *ama-1* in AWC(−) normalized to N2, measured by qPCR. Two-tailed unpaired *t* test with Welch’s correction. *N* = 6 biological replicates. (**F**) OCR in N2, AWC(−), *atfs-1(gk3094)*, and *atfs-1(gk3094)*; AWC(−). Two-tailed unpaired *t* test with Welch’s correction. *N* = 2 biological replicates. (**G**) Log_2_ fold change of the ratio of *mito-1* to *ama-1* in AWC(−), *atfs-1(gk3094)*, and *atfs-1(gk3094)*; AWC(−) normalized to N2, measured by qPCR. Two-tailed unpaired *t* test with Welch’s correction. *N* = 6 biological replicates.

AWC neurons are tonically active in the absence of odorants and become silenced when odorants are presented (*14*). AWC neurons inhibit their downstream interneuron partners; thus, when silenced or ablated, AWC-mediated circuitry is derepressed and mimics an odorant response (*14*). Because AWC ablation has been previously shown to confer pathogen resistance (*12*), we probed whether this phenotype depends on *atfs-1*, the transcription factor that drives UPR^MT^ activation and an innate immune response program (*11*). To ablate AWC neurons, we used a previously developed genetic construct that drives caspase expression under the AWC-specific promoter *ceh-36*, and confirmed that AWC-ablated [AWC(−)] animals were resistant to infection by *P. aeruginosa* compared to wild-type N2 animals [(*12*); Fig. 1A]. We found that AWC(−) animals with an *atfs-1(gk3094)* loss-of-function mutation partially suppressed this resistance (Fig. 1A). We measured ATFS-1 activation in AWC(−) animals using the transcriptional reporter *hsp-6::GFP* (a UPR^MT^ chaperone and target of ATFS-1) (*15*, *16*) in AWC(−) and found that AWC(−); *hsp-6::GFP* animals had pronounced activation of UPR^MT^ compared to wild-type animals (Fig. 1, B and C).

Curious whether the induction of UPR^MT^ downstream of AWC-mediated circuit disinhibition correlated with changes in mitochondrial function, we measured oxygen consumption rates (OCR), a readout of mitochondrial OXPHOS, in N2 versus AWC(−) animals at the last larval stage (L4) (*17*). We assayed these mitochondrial phenotypes at the L4 stage to assess somatic changes in mitochondrial function, before the expansion of the germline, which contributes to the bulk of *C. elegans* mitochondrial content (*18*). We found that AWC(−) animals had markedly reduced OCR at baseline and when treated with the electron transport chain uncoupler FCCP [carbonyl cyanide 4-(trifluoromethoxy)phenylhydrazone] (maximal OCR) (Fig. 1D). The proportional reduction in basal and maximal OCR suggested that AWC(−) animals may have lower mitochondrial mass. To test this, we compared the ratio of a mitochondrial genomic gene (*mito-1*) to a nuclear genomic housekeeping gene (*ama-1*) (*19*, *20*). We found that the ratio of *mito-1* to *ama-1* was significantly reduced in AWC(−) animals compared to N2, indicating a significant reduction in mtDNA (Fig. 1E). We confirmed that the reduction in OCR and mtDNA observed at L4 persists into day 1 (D1) of adulthood, indicating that these phenotypes are not stage specific (fig. S1, A and B).

We assayed whether the reductions of OCR and mtDNA in AWC(−) animals were dependent on ATFS-1 activity and found that *atfs-1(gk3094)*; AWC(−) mutants did not restore OCR and mtDNA content (Fig. 1, F and G). Together, these data suggest that ATFS-1 activation by AWC ablation induces UPR^MT^ and partially protects animals from pathogenic infection but does not seem to play a role in alterations of OCR and reduced mtDNA content. This suggests that a parallel pathway outside of UPR^MT^ might be driving these other mitochondrial phenotypes.

To confirm that reductions in mtDNA correlated with a decrease in mitochondrial content, we used an intestine-specific mitochondrial receptor import subunit TOM-20 reporter (*ges-1p::tomm-20::mKate*) with and without AWC ablation (*21*). We observed reduced fluorescence intensity in AWC(−); *ges-1p::tomm-20::mKate* animals compared to *ges-1p::tomm-20::mKate* alone, indicating a reduction in intestinal mitochondria (fig. S1, C to E). Further, we stained N2 and AWC(−) animals with tetramethylrhodamine ethyl ester (TMRE), a cationic dye that binds active mitochondria, and imaged the distal intestinal region. AWC(−) animals had reduced TMRE staining compared to N2, which indicates that AWC(−) animals have either fewer mitochondria, mitochondria with diminished membrane potential, or both (fig. S1, F and G). These data offer evidence that the reduction of mtDNA in AWC(−) animals correlates with a similar reduction in intestinal mitochondria and TMRE staining intensity.

### Odorant and genetic silencing of AWC neurons activates UPR^MT^, reduces OXPHOS, and depletes mtDNA content

**Fig. 2.**
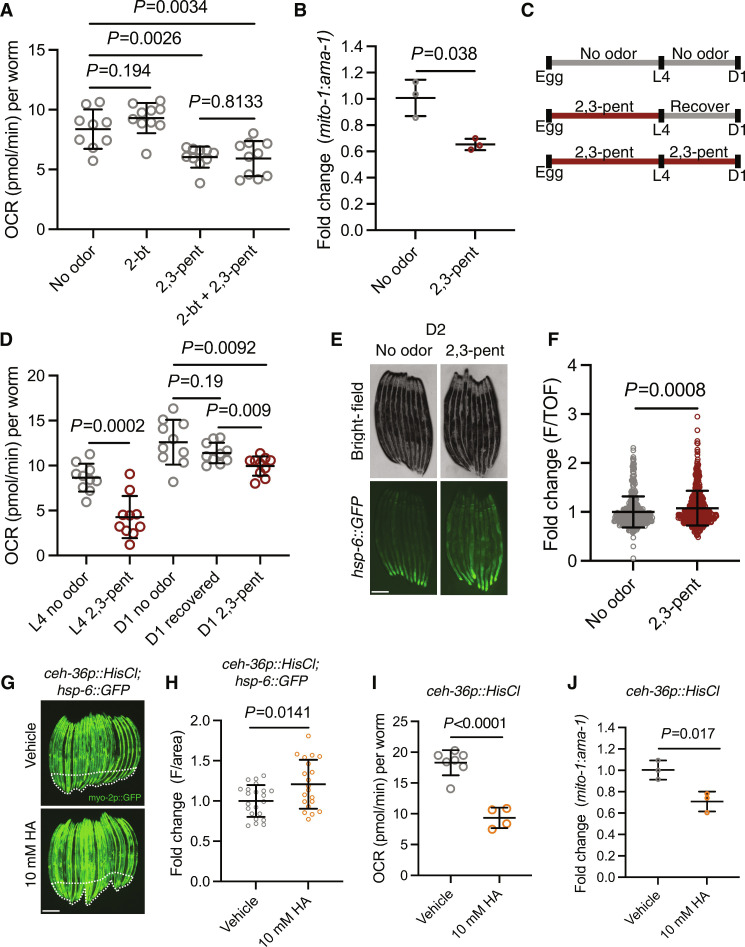
Odorant and genetic silencing of AWC neurons activates UPR^MT^, reduces OXPHOS, and depletes mtDNA content. (**A**) OCR in N2 treated with no odor, 2-butanone (2-bt), 2,3-pentanedione (2,3-pent), and 2-bt and 2,3-pent. Two-tailed unpaired *t* test with Welch’s correction. *N* = 2 biological replicates. (**B**) Log_2_ fold change of the ratio of *mito-1* to *ama-1* in N2 treated with 2,3-pent normalized to untreated N2, measured by qPCR. Two-tailed unpaired *t* test with Welch’s correction. *N* = 3 biological replicates. (**C**) Schematic of recovery assay. (**D**) OCR in control and 2,3-pent treatment at L4, and in control, 24-hour 2,3-pent recovery, and chronic 2,3-pent exposure at D1. Two-tailed unpaired *t* test with Welch’s correction. *N* = 2 biological replicates. (**E**) Representative fluorescent images of *hsp-6::GFP* in no odor– and 2,3-pent–treated animals. Scale bar, 250 μm. (**F**) Fold change of integrated fluorescence intensity (F/TOF) measured by bioSorter of *hsp-6::GFP* in conditions imaged in (E). Two-tailed unpaired *t* test with Welch’s correction. *N* = 2 biological replicates. (**G**) Representative fluorescent images of *ceh-36p::HisCl; hsp-6::GFP* in HA-treated and untreated animals. Body-wall muscle GFP is delineated by dashed lines. Scale bar, 250 μm. (**H**) Fold change of average fluorescence/area of distal intestine (excluding body-wall muscle GFP) imaged in (G), measured by FIJI. Two-tailed unpaired *t* test with Welch’s correction. *N* = 2 biological replicates. (**I**) OCR in HA untreated and HA-treated *ceh-36p::HisCl* animals. Two-tailed unpaired *t* test with Welch’s correction. *N* = 2 biological replicates. (**J**) Log_2_ fold change of the ratio of *mito-1* to *ama-1* in *ceh-36p::HisCl* treated with HA, normalized to untreated *ceh-36p::HisCl*. Two-tailed unpaired *t* test with Welch’s correction. *N* = 3 biological replicates.

Next, we probed whether naturalistic odorant silencing of AWC recapitulates the changes in mitochondrial dynamics achieved through ablation of AWC. AWC neurons develop asymmetrically into two subtypes (AWC^ON^ and AWC^OFF^), which diverge through differential transcriptional control (*22*). These subtypes express shared and distinct odorant receptors, whereby AWC^ON^ is silenced by the volatile bacterial metabolite 2-butanone (2-bt) and AWC^OFF^ is silenced by the volatile bacterial metabolite 2,3-pent (*22*). We exposed N2 wild-type animals to 2-bt, 2,3-pent, and a combination of 2-bt and 2,3-pent and found that 2,3-pent–exposed animals had reduced OCR (Fig. 2A). Notably, there was no combinatorial effect of 2,3-pent and 2-bt on OCR (Fig. 2A), suggesting AWC^OFF^ specificity in driving peripheral OCR reduction. 2,3-pent also significantly decreased mtDNA content compared to untreated N2 animals (Fig. 2B). We tested whether the decrease in OCR due to 2,3-pent–mediated AWC silencing was transient by allowing 2,3-pent–exposed animals to recover from the L4 larval stage to D1 of adulthood (Fig. 2C). We found that 24 hours of recovery from 2,3-pent exposure restored OCR to control levels, and showed significantly higher levels of OCR compared to chronic 2,3-pent exposure (Fig. 2D). These data suggest that AWC^OFF^-mediated circuitry can dynamically regulate peripheral mitochondrial activity in response to a naturalistic cue. 2,3-pent was insufficient to drive induction of UPR^MT^ at D1 (fig. S2, A and B), but mildly induced *hsp-6::GFP* expression from chronic exposure through day 2 (D2) of adulthood (Fig. 2, E and F). We interpreted these results to suggest that silencing AWC^OFF^ by 2,3-pent may not be robust enough to phenocopy UPR^MT^ activation by AWC ablation, despite significantly reducing OCR and mtDNA levels.

To further probe the effects of AWC silencing and to control for any off-target effects of 2,3-pent exposure, we assayed a transgenic mutant that expresses a histamine (HA)–gated chloride channel under the AWC-specific promoter *ceh-36* (*ceh-36p::HisCl)* (*9*). With this strain, HA treatment silences AWC through hyperpolarization, while HA alone has no known endogenous function in *C. elegans* (*9*). We found that *ceh-36p::HisCl; hsp-6::GFP* animals had increased UPR^MT^ activation when treated with HA at D1 (Fig. 2, G and H). This construct contains a *myo-2p::GFP* (body-wall muscle GFP) coinjection marker, so we restricted the *hsp-6::GFP* quantification to the distal intestinal region (Fig. 2, G and H). In addition, HA-treated *ceh-36p::HisCl* animals had reduced OCR and mtDNA (Fig. 2, I and J). Together, these data indicate that odorant and genetic silencing of AWC recapitulates UPR^MT^ activation, reduced OCR, and reduced mtDNA observed by AWC ablation.

### Ablation of AWC neurons remodels peripheral mitochondria dependent on neurotransmission

**Fig. 3.**
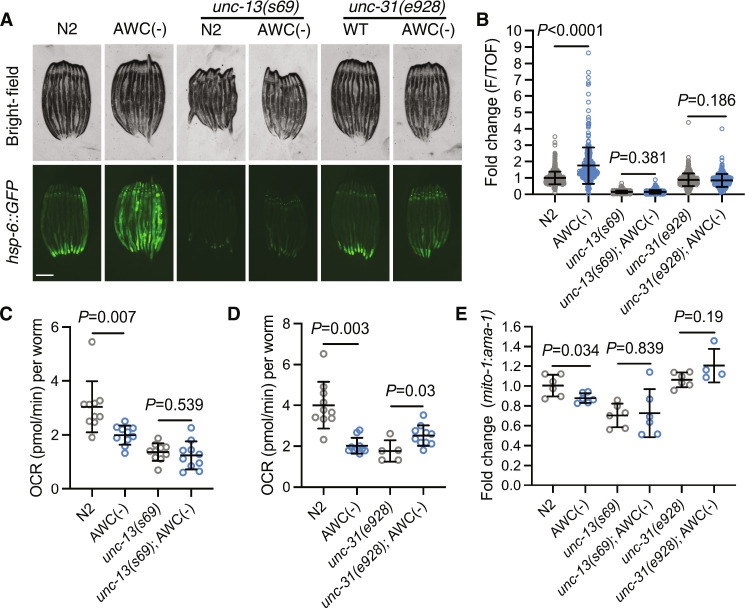
Ablation of AWC neurons remodels peripheral mitochondria dependent on neurotransmission. (**A**) Representative fluorescent images of *hsp-6::GFP* in N2, AWC(−), *unc-13(s69),* AWC(−); *unc-13(s69)*, *unc-31(e928)*, and AWC(−); *unc-31(e928)* animals. Scale bar, 250 μm. (**B**) Fold change of integrated fluorescence intensity (F/TOF) measured by bioSorter of *hsp-6::GFP* in strains imaged in (A). Two-tailed unpaired *t* test with Welch’s correction. *N* = 2 biological replicates. (**C**) OCR in N2, AWC(−), *unc-13(s69)*, and AWC(−); *unc-13(s69)*. Two-tailed unpaired *t* test with Welch’s correction. *N* = 2 biological replicates. (**D**) OCR in N2, AWC(−), *unc-31(e928)*, and AWC(−); *unc-31(e928)* animals. Two-tailed unpaired *t* test with Welch’s correction. *N* = 3 biological replicates. (**E**) Log_2_ fold change of the ratio of *mito-1* to *ama-1* in AWC(−), *unc-13(s69),* AWC(−); *unc-13(s69), unc-31(e928)*, and AWC(−); *unc-31(e928)* animals normalized to N2, measured by qPCR. Two-tailed unpaired *t* test with Welch’s correction. *N* = 4 to 6 biological replicates.

To test whether ablation of AWC remodels mitochondrial function cell nonautonomously, we tested the dependence of the mitochondrial phenotypes on machinery required for neurotransmission. In AWC(−); *hsp-6::GFP* animals, we examined the effect of mutation in *unc-13,* which is required for small clear vesicle (SCV) neurotransmission (*23*), and *unc-31,* which is required for dense core vesicle (DCV) neurotransmission (*24*). We found that loss of either *unc-13* or *unc-31* suppressed peripheral induction of UPR^MT^ in AWC(−); *hsp-6::GFP* reporters (Fig. 3, A and B). Next, we assayed OCR and measured mtDNA in AWC(−); *unc-31(e928)* compared to *unc-31(e938)* mutants, and in AWC(−); *unc-13(s69)* compared to *unc-13(s69)* mutants. We found the reduction in both OCR and mtDNA in AWC(−) *unc-31*–dependent or *unc-13*–dependent neurotransmission (Fig. 3, C to E). These data indicate that ablation of AWC drives SCV- and DCV-dependent signaling to the periphery to induce UPR^MT^, reduce OCR, and reduce mtDNA.

### Ablation of AWC neurons remodels peripheral mitochondria dependent on serotonergic signaling

**Fig. 4.**
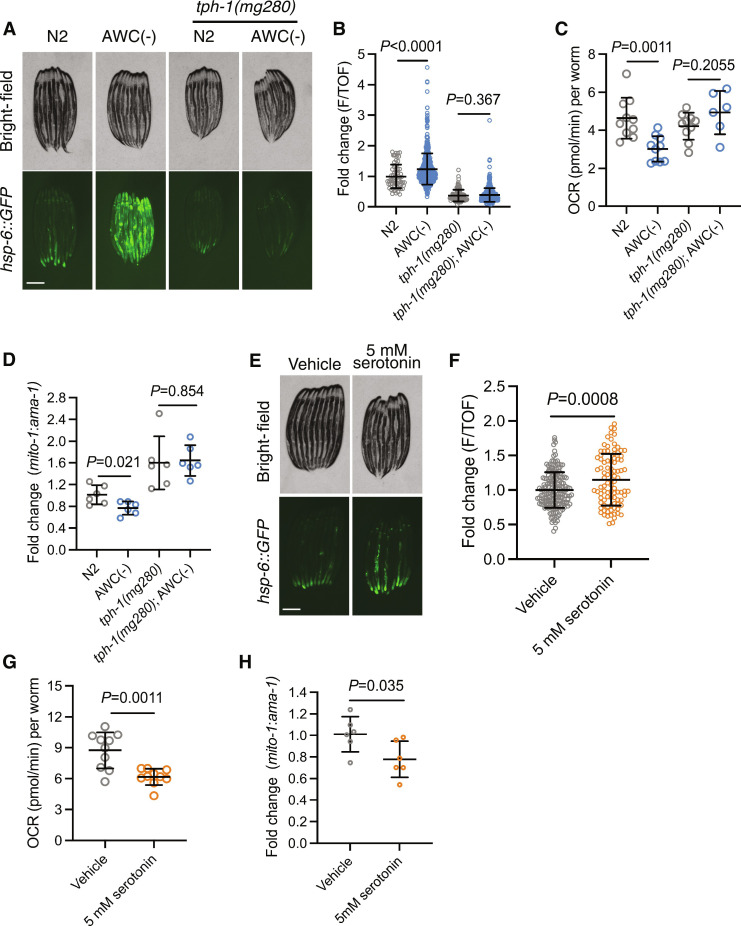
Ablation of AWC neurons remodels peripheral mitochondria dependent on serotonergic signaling. (**A**) Representative fluorescent images of *hsp-6::GFP* in N2, AWC(−), *tph-1(mg280)*, and AWC(−); *tph-1(mg280)* animals. Scale bar, 250 μm. (**B**) Fold change of integrated fluorescence intensity (F/TOF) measured by bioSorter of *hsp-6::GFP* in strains imaged in (A). Two-tailed unpaired *t* test with Welch’s correction. *N* = 2 biological replicates. (**C**) OCR in N2, AWC(−), *tph-1(mg280)*, and AWC(−); *tph-1(mg280)* animals. Two-tailed unpaired *t* test with Welch’s correction. *N* = 2 biological replicates. (**D**) Log_2_ fold change of the ratio of *mito-1* to *ama-1* in AWC(−), *tph-1(mg280)*, and AWC(−); *tph-1(mg280)* animals normalized to N2, measured by qPCR. Two-tailed unpaired *t* test with Welch’s correction. *N* = 6 biological replicates. (**E**) Representative fluorescent images of *hsp-6::GFP* in N2 treated with vehicle or 5 mM serotonin. Scale bar, 250 μm. (**F**) Fold change of average fluorescence/area of conditions imaged in (E), measured by FIJI. Two-tailed unpaired *t* test with Welch’s correction. *N* = 2 biological replicates. (**G**) OCR in N2 treated with vehicle or 5 mM serotonin. Two-tailed unpaired *t* test with Welch’s correction. *N* = 2 biological replicates. (**H**) Log_2_ fold change of the ratio of *mito-1* to *ama-1* in N2 treated with 5 mM serotonin normalized to vehicle-treated N2, measured by qPCR. Two-tailed unpaired *t* test with Welch’s correction. *N* = 6 biological replicates.

Having determined that AWC ablation remodels peripheral mitochondrial dynamics nonautonomously, we investigated which specific molecule(s) carried by either SCVs or DCVs propagates this signal. SCVs carry canonical neurotransmitters and biogenic amines, while DCVs also carry biogenic amines and neuropeptides. The biogenic amine serotonin, transmitted in both SCVs and DCVs, has previously been established as a mitokine (*4*, *5*, *25*). Thus, we tested whether loss of *tph-1*, the gene required for the synthesis of serotonin, was required for AWC-mediated UPR^MT^ induction. AWC(−) animals with a *tph-1(mg280)* mutation completely suppressed UPR^MT^ induction (Fig. 4, A and B). Next, we measured OCR and mtDNA in AWC(−); *tph-1(mg280)* compared to *tph-1(mg280)* mutants and found that loss of serotonin signaling suppressed the reductions in OCR and mtDNA (Fig. 4, C and D). These data suggest that upon ablation of AWC, AWC-regulated circuitry becomes disinhibited and remodels peripheral mitochondria dependent on intermediate serotonergic neuron(s).

Demonstrating the requirement of serotonin for the changes in mitochondrial dynamics in AWC(−) animals, we examined whether exogenous serotonin treatment was sufficient to recapitulate these phenotypes. We treated *hsp-6::GFP* animals with exogenous serotonin, which was sufficient to induce UPR^MT^, albeit mildly compared to AWC(−) animals (Fig. 4, E and F). We then treated N2 animals with exogenous serotonin and measured OCR and mtDNA levels. We found that serotonin treatment was also sufficient in reducing OCR and mtDNA (Fig. 4, G and H).

To more directly test whether serotonin signaling regulates mitochondrial homeostasis in recipient cells, we supplemented serotonin to cultured human BJ fibroblasts, a karyotypically stable cell line that naturally expresses multiple serotonin receptors (*26*). Consistent with what we observed in our *C. elegans* model, cells treated with 10 μM serotonin had a reduced trend in MitoTracker Green staining, and cells treated with 100 μM serotonin had significantly reduced MitoTracker Green staining, indicative of reduced mitochondrial mass (fig. S3A). Moreover, 10 and 100 μM serotonin treatment reduced MitoSOX Red staining, indicating that the generation of mitochondrial reactive oxygen species (ROS) and overall mitochondrial electron transport chain activity is reduced (fig. S3B). We confirmed the MitoSOX Red readout by measuring OCR and observed a significant reduction in OCR by 10 and 100 μM serotonin treatment (fig. S3C). We also performed quantitative polymerase chain reaction (qPCR) on *Atf3*, a stress-induced transcription factor (*27*), *Atf4*, a transcriptional regulator of the integrated stress response (ISR) (*28*), and *Atf5*, a transcriptional regulator of human UPR/UPR^MT^ (*29*), to determine whether serotonin-induced changes in mitochondrial mass and function correlated with activation of human ISR/UPR^MT^. We found that 10 and 100 μM serotonin treatment failed to up-regulate these transcription factors (fig. S3D), and that tested expression of *Atf4* and *Atf5* targets was also unaffected by either concentration of serotonin treatment (fig. S3E) (*30*–*32*). Together, these data show that serotonin treatment is sufficient to reduce mitochondrial mass and activity in human fibroblasts, recapitulating our observations in *C. elegans.* Strikingly, however, these phenotypes are uncoupled from ISR/UPR^MT^ activation in human fibroblasts, whereas UPR^MT^ activation and mitochondrial dysfunction are correlated in *C. elegans* downstream serotonergic activity. These data demonstrate that serotonin can directly act on cells to reduce mitochondrial mass and activity and may have a conserved role in regulating mitochondrial function across species.

### Ablation of AWC activates UPR^MT^ partially dependent on PDR-1 and reduces OCR and mtDNA levels fully dependent on PDR-1

**Fig. 5.**
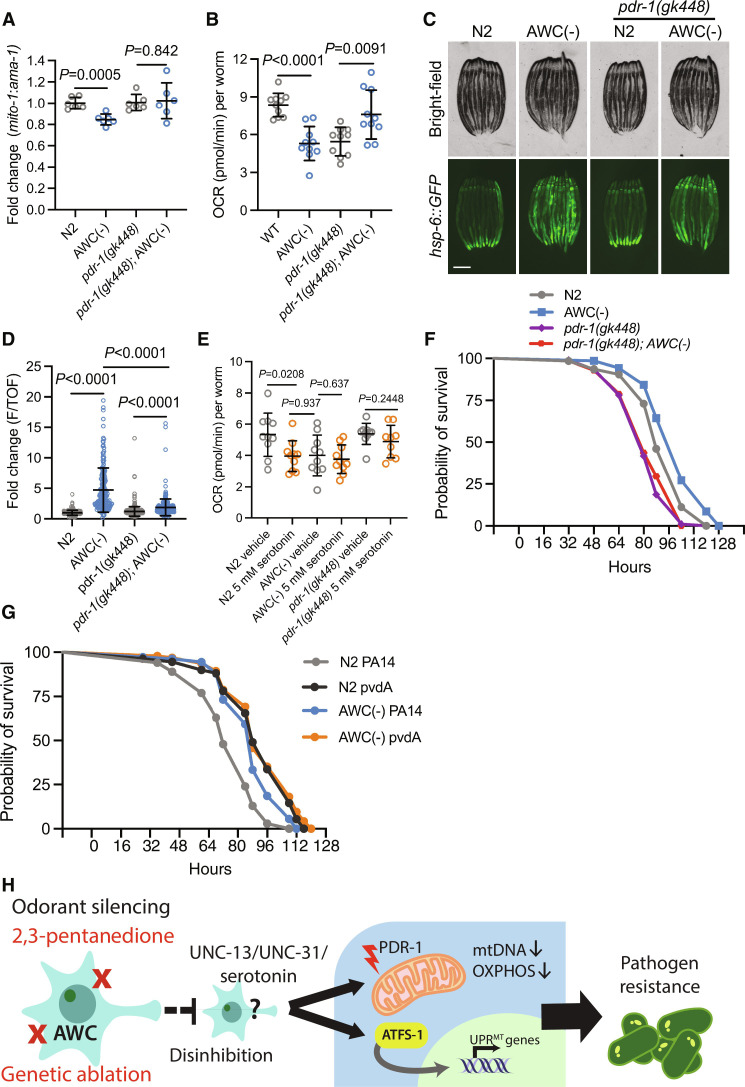
Ablation of AWC activates UPR^MT^ partially dependent on PDR-1 and reduces OCR and mtDNA levels fully dependent on PDR-1. (**A**) Log_2_ fold change of the ratio of *mito-1* to *ama-1* in AWC(−), *pdr-1(gk448)*, and *pdr-1(gk448)*; AWC(−) animals normalized to N2, measured by qPCR. Two-tailed unpaired *t* test with Welch’s correction. *N* = 6 biological replicates. (**B**) OCR in N2, AWC(−), *pdr-1(gk448)*, and *pdr-1(gk448)*; AWC(−) animals. Two-tailed unpaired *t* test with Welch’s correction. *N* = 3 biological replicates. (**C**) Representative fluorescent images of *hsp-6::GFP* in N2, AWC(−), *pdr-1(gk448)*, and *pdr-1(gk448)*; AWC(−) animals. Scale bar, 250 μm. (**D**) Fold change of integrated fluorescence intensity (F/TOF) measured by bioSorter of *hsp-6::GFP* in strains imaged in (C). Two-tailed unpaired *t* test with Welch’s correction. *N* = 2 biological replicates. (**E**) OCR in N2, AWC(−), and *pdr-1(gk448)* treated with vehicle or 5 mM serotonin. Welch’s correction. *N* = 2 biological replicates. (**F**) Survival of N2, AWC(−), *pdr-1(gk448)*, and *pdr-1(gk448)*; AWC(−) animals on PA14. Log-rank Mantel-Cox test: N2 versus AWC(−) (*P* = 0.0028); *pdr-1(gk448) versus pdr-1(gk448)*; AWC(−) (*P* = 0.9068); AWC(−) versus *pdr-1(gk448)*; AWC(−) (*P* < 0.0001). *N* = 2 biological replicates. (**G**) Survival of N2 and AWC(−) on PA14 and PA14 mutant *pvdA*. Log-rank Mantel-Cox test: N2 PA14 versus AWC(−) PA14 (*P* < 0.0001); N2 *pvdA* versus AWC(−) *pvdA* (*P* = 0.3590); AWC(−) PA14 versus AWC(−) *pvdA* (*P* = 0.1129). *N* = 2 biological replicates. (**H**) Summary schematic.

Observing that AWC(−) animals have activated UPR^MT^, but reduced mtDNA and OCR independent of *atfs-1*, we asked if an additional mitochondrial quality control pathway might be induced by the loss of AWC. Therefore, we asked whether the reduced OCR and mtDNA phenotypes were driven by active clearance of mitochondria. To test this, we measured whether these mitochondrial phenotypes depend on mitophagy machinery *pdr-1* (parkin homolog). First, we compared mtDNA in AWC(−) to AWC(−); *pdr-1(gk448)* mutants and observed a rescue in mtDNA to N2 levels (Fig. 5A). Next, we observed a rescue in OCR in AWC(−); *pdr-1(gk448)* compared to AWC(−) mutants (Fig. 5B). Finally, we measured *hsp-6::GFP* expression in AWC(−) animals with a *pdr-1(gk448)* mutation and observed a partial suppression of UPR^MT^ induction (Fig. 5, C and D). In addition, we measured NDUFS3, a mitochondrial ETC complex 1 protein, in N2 and AWC(−) animals with and without *pdr-1* mutation. We found that NDUFS3 trends down in AWC(−) animals but shows no difference in *pdr-1(gk448)* mutants and *pdr-1(gk448)*; AWC(−) mutants (fig. S4, A and B). Together, these data show that *pdr-1* is completely required for the OCR and mtDNA phenotypes of AWC(−) animals but appears to play a less important role for the induction of UPR^MT^.

To understand if serotonin signaling reduces OCR dependent on *pdr-1*, we treated N2, AWC(−), and *pdr-1(gk448)* mutants with exogenous serotonin. We observed that serotonin treatment failed to further reduce OCR in AWC(−) mutants, and that serotonin failed to reduce OCR in *pdr-1(gk448)* mutants (Fig. 5E). These data indicate that PDR-1 functions downstream of serotonin activity to reduce OCR, and that AWC(−) mutants may experience a floor effect, whereby additional serotonergic activity fails to further diminish OCR.

Finally, we aimed to explore the physiological relevance for linking olfaction to peripheral mitophagy. Mitophagy has been identified as a prosurvival strategy during infection by *P. aeruginosa*, and mutants lacking mitophagy machinery are more sensitive to *P. aeruginosa* infection (*33*). In an effort to proliferate, *P. aeruginosa* chelates rate-limiting iron from host mitochondria by a family of siderophores called pyoverdines (*34*). In response to pyoverdine, *C. elegans* perform mitophagy to limit *P. aeruginosa* virulence (*19*). We performed a PA14 survival assay on N2, AWC(−), *pdr-1(gk448)*, and *pdr-1(gk448)*; AWC(−) strains and found that the resistance phenotype observed in AWC(−) animals fully depends on *pdr-1* (Fig. 5F). Next, we tested whether resistance to *P. aeruginosa* pyoverdine contributed to the AWC(−) pathogen resistance phenotype. To do this, we performed a pathogen resistance assay using N2 and AWC(−) animals on wild-type *P. aeruginosa* strain PA14 and on mutant PA14 strain *pvdA* (*35*), which is deficient in pyoverdine biosynthesis (*34*). We found that N2 animals lived longer on *pvdA* compared to PA14 and lived equally long as AWC(−) animals on *pvdA* (Fig. 5G). Further, there was no difference in pathogen resistance between AWC(−) animals on PA14 compared to AWC(−) animals on *pvdA* (Fig. 5G). The lack of synthetic pathogen resistance in AWC(−) on *pvdA* suggests that the resistance phenotype results from their resistance to iron chelation.

## DISCUSSION

We have identified a unique role for AWC olfactory neurons in regulating peripheral mitochondrial dynamics. AWC(−) animals exhibit induction of UPR^MT^, reduction in OXPHOS, and a depletion of mtDNA dependent on serotonergic signaling (Figs. 1 and 3). These data fit with previous studies that established serotonin as a mitokine, where serotonergic signaling was required for UPR^MT^ activation downstream of neuronal mitochondrial dysfunction (*4*, *5*, *25*). Here, however, we identify that an environmental cue (bacterial metabolite 2,3-pent) and a sensory modality (olfaction) can drive nonautonomous regulation of peripheral mitochondrial dynamics absent of genetically driven neuronal mitochondrial perturbation (Fig. 2).

2,3-pent is a metabolite produced by bacteria, including the pathogen *P. aeruginosa* (*13*). We speculate that the olfactory nervous system may have evolved a unique role in relaying bacterial odorant cues, like 2,3-pent, to prepare the periphery for potential homeostatic perturbations, such as metabolic stress or pathogenic infection. We show that AWC(−) animals are resistant to infection from *P. aeruginosa* strain PA14 partially dependent on *atfs-1* (Fig. 1) and fully dependent on *pdr-1* (Fig. 5). Further, AWC(−) animals show no additional benefit on PA14 mutant *pvdA*, which lacks iron chelator pyoverdine synthesis, suggesting that AWC(−) animals are long lived on PA14 due to resistance to pyoverdine (Fig. 5). With these data, we hypothesize that olfactory neurons may couple bacterial odorant cues to mitophagy as an anticipatory strategy to resist pathogenic insult. Implicating olfaction as an upstream-most coordinator of peripheral UPR^MT^ activation and mitochondrial quality control grounds this biological phenomenon in an evolutionary context.

In addition, we show that exogenous serotonin treatment is sufficient to drive UPR^MT^, reduce OXPHOS, and reduce mtDNA, phenocopying AWC ablation (Fig. 3). This suggests that AWC ablation may drive these mitochondrial phenotypes through disinhibited serotonin signaling. We demonstrate that serotonin may play a conserved role in mitochondrial regulation in mammalian cells, whereby serotonin treatment reduces mitochondrial mass and activity in mammalian fibroblasts (fig. S2). Conversely, we observed that loss of serotonin neurotransmission by *tph-1* mutation increases mtDNA copy number in wild-type N2 animals (Fig. 4D). These data suggest that serotonin not only is sufficient to drive mitochondrial depletion in recipient tissues but also may be required to maintain appropriate mitochondrial mass in wild-type animals and presents an attractive avenue for further study. In sum, our data indicate a model whereby olfaction can signal to the periphery to alter mitochondrial stress responses, such as UPR^MT^ by activation of *atfs-1*, and mitochondrial quality control pathways, such as mitophagy.

While we have provided a naturalistic rationale for why the nervous system seeks to control peripheral mitochondrial quality, it is yet unclear how the nervous system mechanistically regulates mitophagy in peripheral tissues. Mitophagy is a complex process regulated at the transcriptional level (*20*) and through posttranslational modification (*36*). Future studies should aim to understand how peripheral tissues respond to the signals from the nervous system, such as serotonergic signals, to coordinate mitophagy.

In the broader context, there is mammalian evidence that neuropeptidergic afferents to the mesentery can drive the release of serotonin from mast cells, which store large quantities of the neurotransmitter (*37*). In the intestinal milieu, serotonin is an important signaling molecule that drives immune cell activation and the release of proinflammatory cytokines, increases cytotoxicity, and is important for maintaining microbiome homeostasis (*38*). Therefore, our results warrant further investigation into whether an olfactory-to-intestine signaling axis may be conserved in mammals such that olfactory activation may increase intestinal serotonin availability and increased immune activation in response to environmental cues.

## MATERIALS AND METHODS

### *C. elegans* strains and details

Nematodes were maintained at 15° or 20°C on standard nematode growth medium (NGM) agar plates seeded with *Escherichia coli* strain OP50. All experiments were conducted on bleach synchronized populations at 20°C on OP50 plates. Synchronization was achieved by washing animals fed with OP50 with M9 solution (22 mM KH_2_PO_4_ monobasic, 42.3 mM Na_2_HPO_4_, 85.6 mM NaCl, and 1 mM MgSO_4_), bleached using a solution of 1.8% sodium hypochlorite and 0.375 M KOH diluted in water, for 4 to 5 min. Intact eggs were then washed three times with M9 solution, and intact eggs were verified under the microscope after seeding. See table S1 for a complete list of *C. elegans* strains used in this study.

### Microscopy and quantification of *hsp-6::GFP* and *ges-1p::tomm-20::mKate*

Imaging of strains in the *hsp-6::GFP* and *ges-1p::tomm-20::mKate* backgrounds were performed as previously described (*24*). Briefly, strains were grown from egg to day 1 of adulthood (D1), unless otherwise specified, at 20°C and picked in bright field to avoid biasing reporter intensity. Animals were paralyzed with 10 μl of 100 mM sodium azide for *hsp-6::GFP* experiments and 5 mM levamisole for *ges-1p::tomm-20::mKate* experiments. Animals were oriented from head to tail on unseeded NGM plates. Images were captured on a Leica M250FA stereoscope with a Hamamatsu ORCA-ER camera with LAS-X software. Bright-field and fluorescent images were taken with experiment-matched exposure time and laser intensity with a 1× objective and 4× magnification.

Each microscopy experiment was sampled from a population that was subsequently quantified using a large-particle flow cytometer (Union Biometrica bioSorter) as previously described (*24*). Data for time of flight (TOF; a measure of length), extinction (width), and integrated GFP fluorescence were collected. Data were gated by TOF and extinction to exclude eggs or any larvae present. Data are presented as integrated fluorescence divided by TOF, to normalize for varying lengths (F/TOF) in arbitrary units, normalized to control. The experiments using *Ex[ceh-36p::HisCl; myo-2p::GFP]; hsp-6::GFP* were quantified by dividing distal intestinal fluorescence intensity (excluding body-wall muscle GFP)/area, and the experiments using *ges-1p::tomm-20::mKate* were quantified by dividing intestinal fluorescence intensity [excluding coelomocyte red fluorescent protein (RFP)]/area using Fiji (Fiji Is Just ImageJ, NIH) software. High-magnification images of strains in the *ges-1p::tomm-20::mKate* background (fig. S1C) were taken at the distal intestinal region using a THUNDER Imager Compound Microscope (Leica) with a 63× objective.

### Mitochondrial OCR measurement

OCR in *C. elegans* was measured using a Seahorse XeF96 (Agilent) instrument using the extracellular flux assay kit (Agilent). Twenty to 40 L4 animals were plated in 200 μl of M9 per well, with 5 to 10 wells per strain. Basal OCR was read using the following program: five cycles of 2 min mix, 30 s wait, 2 min measure. For maximal oxygen consumption, 22 μl of 100 mM FCCP (Sigma-Aldrich) was injected into each well, and OCR was measured using the following program: nine cycles of 2 min mix, 30 s wait, 2 min measure. All assays ended with sodium azide (NaN_3_) treatment to measure nonmitochondrial respiration and to paralyze the animals for quantification. Twenty-two microliters of 400 mM NaN_3_ was injected into each well and measured using the following program: four cycles of 2 min mix, 30 s wait, and 2 min measure. Following the assay, paralyzed animals were quantified per well. In OCR figures, each data point represents the average OCR in pmol per minute per the quantity of animals in each well.

OCR in BJ fibroblasts was measured using a Seahorse XeF96 Analyzer (Agilent) according to the manufacturer’s protocol. In brief, cells were seeded in a Seahorse XFe96 Cell Culture Microplate (34222, Agilent) and grown to 60 to 70% confluency at the time of measurement. The Seahorse XF Dulbecco’s modified Eagle’s medium (DMEM) (103575-100, Agilent) supplemented with glucose (4.5 g/liter), 1 mM sodium pyruvate, GlutaMAX, nonessential amino acids (NEAAs), and antibiotics [but excluding fetal bovine serum (FBS)] was substituted for regular culture medium 1 hour before measurement in a 37°C CO_2_-free incubator. OCR was measured using the following program: nine cycles of 2 min mix, 30 s wait, 2 min measure.

### mtDNA quantification

mtDNA was quantified by qPCR as previously described (*19*, *20*). Briefly, 20 to 30 egg-lay synchronized L4 animals were collected in 20 μl of lysis buffer [50 mM KCl, 10 mM tris-HCl (pH 8.3), 2.5 mM MgCl_2_, 0.45% NP-40, 0.45% Tween 20, 0.01% gelatin, with freshly added proteinase K (200 μg/ml)] and flash-frozen at −80°C for at least 20 min before lysis. Lysis was performed at 65°C for 80 min. Each reaction was done in triplicate using 1 μl of mtDNA lysate, with SYBR Select Master Mix (Thermo Fisher Scientific). The following mtDNA-specific primers were used: *mito-1*: F′ gtttatgctgctgtagcgtg, R′ ctgttaaagcaagtggacgag. The following genomic DNA–specific primers were used: *ama-1*: F′ tggaactctggagtcacacc, R′ catcctccttcattgaacgg. Data presented are ratios of mtDNA to genomic DNA (*mito-1*:*ama-1*) normalized to N2 values.

### TMRE staining and quantification

D1 strains were incubated in 200 μl of S Basal supplemented with TMRE (T699, Thermo Fisher Scientific) to a final concentration of 10 μM for 45 min on a gentle shaker at room temperature, covered in foil. Excess dye was washed off using S Basal 2×. Animals were destained of excess TMRE by transferring onto OP50-seeded NGM plates for 30 min before imaging.

High-magnification images of strains stained with TMRE (fig. S1F) were taken at the distal intestinal region using a THUNDER Imager Compound Microscope (Leica) with a 63× objective. TMRE experiments were quantified by dividing fluorescence intensity/area using Fiji (Fiji Is Just ImageJ, NIH) software.

### Odorant exposure

Bleach synchronized eggs were seeded onto OP50 plates. For odorant conditions, 1 μl of undiluted odorant was applied to the lid of the plate and inverted. Odorant-exposed plates were placed in a 3 × 5–inch airtight box, in a fume hood at room temperature. Control condition animals were placed in an identical box without odors in the same fume hood. Odors were refreshed every 24 hours. Odorants used were 2,3-pent (241962, Sigma-Aldrich) and 2-bt (110264, Sigma-Aldrich).

### Pharmacological treatments in *C. elegans*

To inhibit germline proliferation, L4 populations were washed off plates in M9 and seeded onto pretreated (+)-5-fluorodeoxyuridine (FUDR; Spectrum Chemical) OP50 plates. Plates were pretreated with 100 μl of FUDR (10 mg/ml) and allowed to dry before L4 animals were plated.

For assays using the AWC-specific HA-gated chloride channel construct [*Ex[ceh-36::HisCl;myo-2::GFP]*, a gift from the T. Hoppe laboratory (*9*)], OP50 plates were treated with 500 μl of 200 mM HA (Sigma-Aldrich) dissolved in water, for a final plate concentration of 10 mM. L4 *Ex[ceh-36::HisCl;myo-2::GFP]* animals were transferred to dried HA or vehicle-treated plates and assayed at D1.

For exogenous serotonin treatment, OP50 plates were treated with 330 μl of 150 mM serotonin hydrochloride (H9523, Sigma-Aldrich), dissolved in water, for a final plate concentration of 5 mM. Synchronized eggs were plated onto dried vehicle-treated or serotonin-treated plates. mtDNA quantification and OCR for serotonin-treated animals were performed at L4, while *hsp-6::GFP* imaging was performed at D1 of adulthood.

### Cell culture

Human BJ fibroblasts were obtained from the American Type Culture Collection (BJ-5ta) and grown in DMEM (11995, Thermo Fisher Scientific) supplemented with 2 mM GlutaMAX (35050, Thermo Fisher Scientific), 10% FBS (VWR), NEAAs (100 X, 11140, Thermo Fisher Scientific), and penicillin-streptomycin (100 X, 15070, Thermo Fisher Scientific) in 5% CO_2_ at 37°C. The identity of cell lines was confirmed through their STR profiling by UC Berkeley DNA Sequencing Facility. Mycoplasma-negative status was confirmed by PCR Detection Kit.

### Serotonin treatment in BJ fibroblasts

Serotonin hydrochloride (H9523, Sigma-Aldrich) was dissolved in 1× phosphate-buffered saline (PBS) and supplemented to cell culture medium at 10 and 100 μM final concentrations. Cells were treated for 24 hours before analysis.

### Quantitative reverse transcription PCR

RNA was purified from vehicle- and serotonin-treated BJ fibroblasts using an RNeasy Mini Kit (74106, Qiagen) and stored at −80°C. cDNA was synthesized using a QuantiTect Reverse Transcription Kit (205311, Qiagen). qPCR was performed using the target sequences listed in table S2.

### Flow cytometry

Cells were plated onto culture plates (Corning) and allowed to grow overnight. Drugs were directly added to medium. Cells (should be around 80% confluency) were harvested after 24 hours of serotonin treatment. To perform flow cytometry staining, cells were trypsinized and stained with 150 nM MitoTracker Green (M7514, Thermo Fisher Scientific) or 5 μM MitoSOX Red (M36008, Thermo Fisher Scientific) in culture medium at 37°C for 30 min. After staining, cells were washed once with cold culture medium and resuspended in cold fluorescence-activated cell sorting (FACS) buffer (PBS with 0.1% bovine serum albumin and 2 mM EDTA). Samples were then analyzed with a four-laser Attune NxT flow cytometer (Thermo Fisher Scientific). Acquired data were analyzed using FlowJo 10.

### *P. aeruginosa* resistance assay

*P. aeruginosa* strain PA14 was cultured at 37°C in LB overnight before spotting 20 μl of inoculum onto slow killing plates. *P. aeruginosa* strain *pvdA* was cultured at 37°C overnight in LB supplemented with gentamicin (15 μg/ml) before spotting onto slow killing plates supplemented with gentamicin (15 μg/ml) (*35*). Inoculum was spread to cover >75% of the plate. Seeded plates were cultured at 37°C overnight. PA14 and *pvdA* plates were allowed to come to room temperature before treatment with FUDR as previously described. FUDR was used to prevent pathogen-induced in utero egg hatching. Synchronized L4 animals were transferred to PA14 plates, with 10 to 15 animals on eight plates per genotype. Lethality was determined by the absence of movement after a nose and tail prod. Lethality was measured each day, with more frequent measurements as survival decreased.

### 2,3-pent odorant recovery assay

Synchronized eggs were plated on OP50 and treated with 2,3-pent or no odorant as described. Baseline OCR measurements were performed at L4 stage. 2,3-pent–treated L4 animals were washed off plates using M9 and washed three times with M9 before replating on a fresh OP50 plate. 2,3-pent–exposed animals were allowed to recover without odor for 24 hours. OCR was performed on 2,3-pent–recovered animals, no odor–exposed animals, and continuously 2,3-pent–exposed animals at D1 of adulthood.

### Western blot analysis

Around 1000 *C. elegans* per strain were grown on OP50 to the L4 stage and were washed off in M9 before an additional M9 wash. Animals were lysed by sonicating (Qsonica) in ice-cold radioimmunoprecipitation assay lysis buffer containing proteinase inhibitors (78439, Thermo Fisher Scientific). Dithiothreitol was added to protein lysate to a final concentration of 100 mM. Protein lysate concentration was quantified by Qubit Protein Broad Range Assay (Q33211, Thermo Fisher Scientific) using a Qubit 4 Fluorometer (Thermo Fisher Scientific), before loading onto tris-glycine 4 to 12% gels (Invitrogen). Gels were transferred using NuPAGE transfer buffer (Invitrogen) on nitrocellulose membranes (Bio-Rad). Membranes were blocked with LI-COR PBS Blocking Buffer (LI-COR) with Tween. Membranes were probed with antibodies targeted to NDUFS3 (ab14711, Abcam) and α-tubulin (T9026, Sigma). Membranes were imaged using the Odyssey CLx Imaging System (LI-COR) and analyzed using Image Studio (LI-COR).

### Statistical analyses

Statistical analysis was performed using GraphPad Prism 9.2.0. Individual analyses are as described in figure legends. Lifespans were analyzed using a log-rank Mantel-Cox test. Two condition comparisons were analyzed using two-tailed unpaired Welch’s *t* tests.
