# Supplementary material for: Olfaction regulates peripheral mitophagy and mitochondrial function
Source: Sci Adv. 2024 Jun 21;10(25):eadn0014. doi: 10.1126/sciadv.adn0014 (PMC11192085; doi:10.1126/sciadv.adn0014)
Supplement: Supplementary file 1 — Figs. S1 to S4 Tables S1 and S2 [file sciadv.adn0014_sm.pdf]

Science Advances
AAAS

# Supplementary Materials for

## Olfaction regulates peripheral mitophagy and mitochondrial function

Julian G. Dishart *et al.*

Corresponding author: Andrew Dillin, dillin@berkeley.edu

*Sci. Adv.* **10**, eadn0014 (2024)
DOI: 10.1126/sciadv.adn0014

**This PDF file includes:**

Figs. S1 to S4
Tables S1 and S2

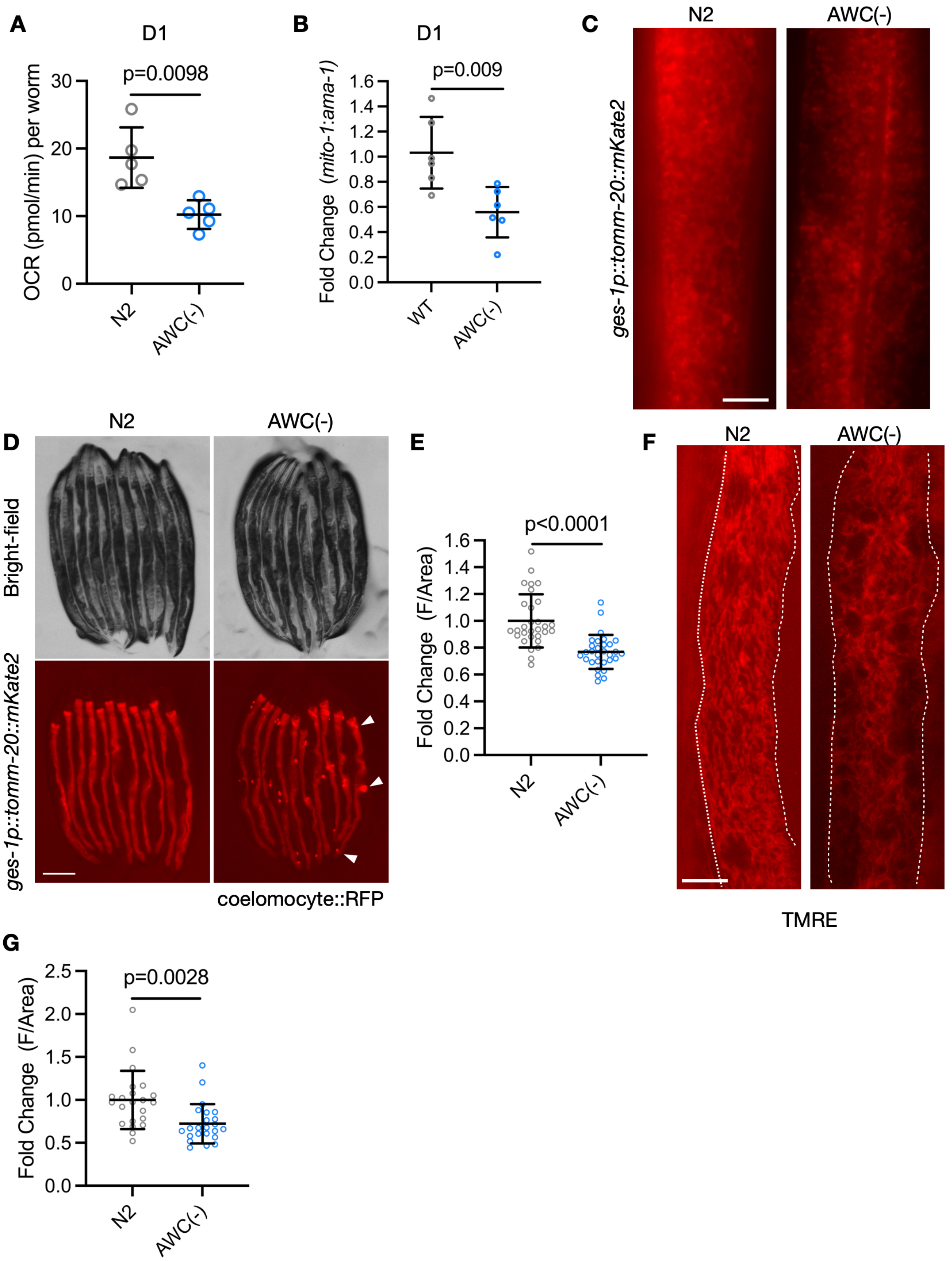


**Supplementary Figure 1: Visualization of mitochondria confirm mtDNA depletion in AWC(-) animals.**

(**A**) Oxygen consumption rate (OCR) in N2 and AWC(-) at day 1 (D1) of adulthood. Two-tailed unpaired t test with Welch's correction. N=2 biological replicates. (**B**) Log2 fold change of the ratio of *mito-1* to *ama-1* in AWC(-) normalized to N2, measured by qPCR at D1. Two-tailed unpaired t test with Welch's correction. N=6 biological replicates. (**C**) High magnification (63x) images of *ges-1p::tomm-20::mKate* and AWC(-); *ges-1p::tomm-20::mKate*. Scale bar = 20 µm. (**D**)

Representative fluorescent images of *ges-1p::tomm-20::mKate and AWC(-); ges-1p::tomm-20::mKate* animals. Scale bar = 250 µM. (**E**) Fold change of average fluorescence / area of whole intestine, excluding coelomocyte RFP (marked by arrows) imaged in panel D, measured by FIJI. Two-tailed unpaired t test with Welch's correction. N=2 biological replicates. (**F**) High magnification (63x) images of N2 and AWC(-) animals stained with TMRE. Scale bar = 20 µM. (**G**) Fold change of average fluorescence / area of intestine (quantification area delineated in panel F), measured by FIJI. Two-tailed unpaired t test with Welch's correction. N=2 biological replicates.

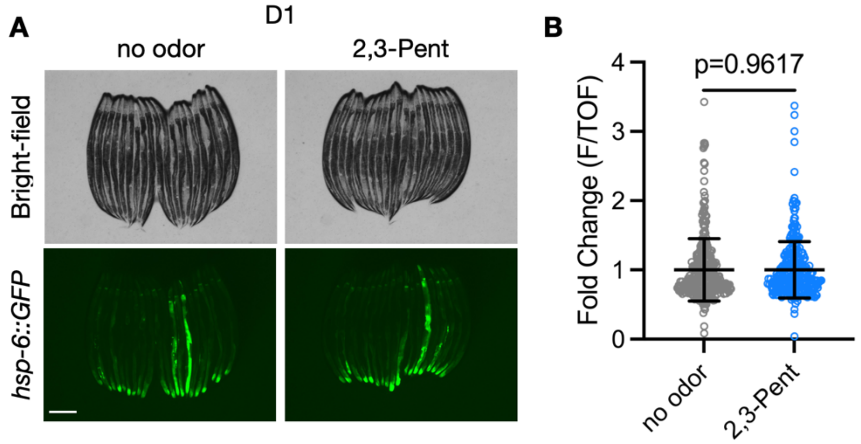


**Supplementary Figure 2: Chronic 2,3-pentanedione exposure fails to induce mtUPR at day 1 of adulthood.**

(**A**) Representative fluorescent images of *hsp-6::GFP* in N2 without odor and with 2,3-pentanedione (2,3-pent) at D1 of adulthood. Scale bar = 250 μM. (**B**) Fold change of integrated fluorescence intensity [fluorescence (F) / time of flight (TOF)] measured by bioSorter of *hsp-6::GFP* in conditions imaged in panel A. Two-tailed unpaired t test with Welch's correction. N=2 biological replicates.

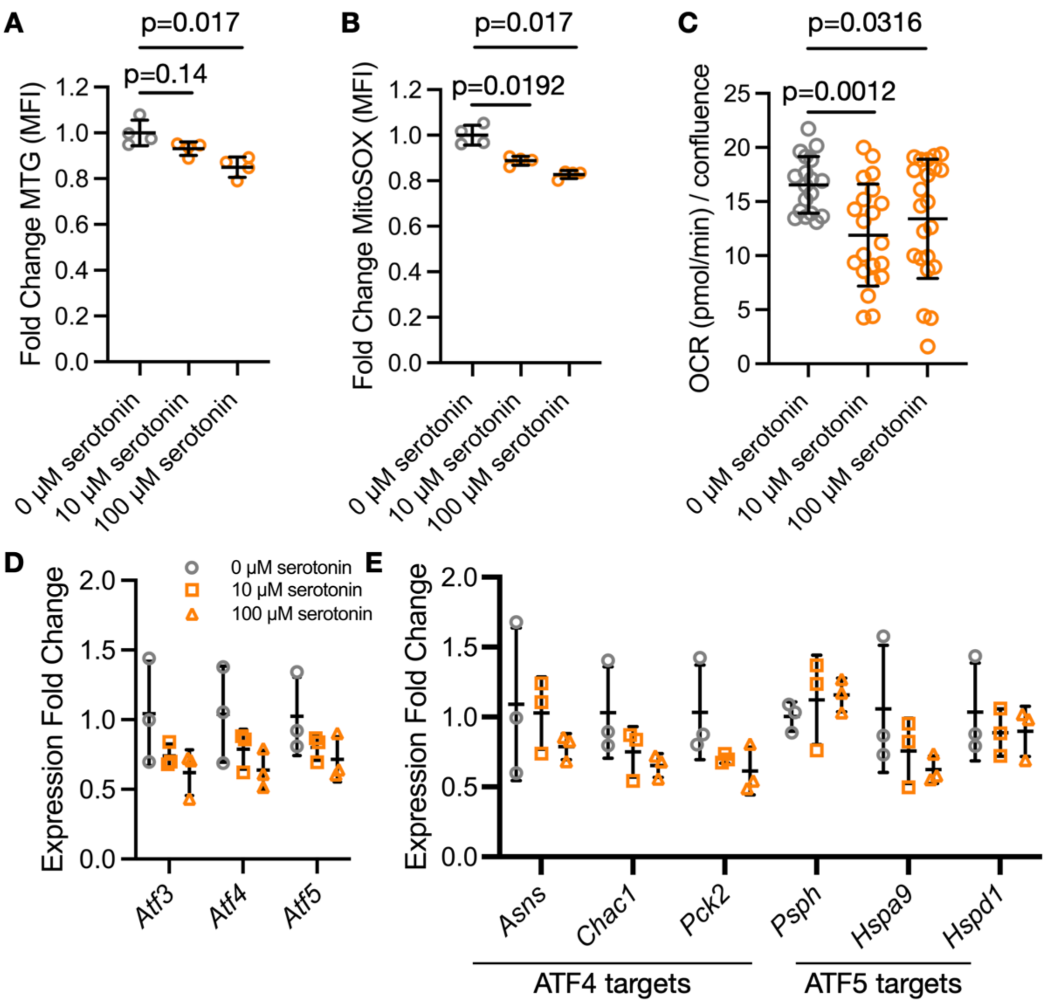


**Supplementary Figure 3. Serotonin treatment reduces mitochondrial mass and activity in cultured human fibroblasts.**

(**A**) Fold change of mean fluorescence intensity (MFI) of human BJ fibroblasts stained with MitoTracker Green (MTG) with vehicle, 10 µM and 100 µM serotonin treatments. Two-tailed unpaired t test with Welch's correction. N=4 biological replicates. (**B**) Fold change of mean fluorescence intensity (MFI) of human BJ fibroblasts stained with MitoSOX with vehicle, 10 µM and 100 µM serotonin treatments. Two-tailed unpaired t test with Welch's correction. N=4 biological replicates. (**C**) OCR in BJ fibroblasts treated with vehicle, 10 µM and 100 µM serotonin. N=3 biological replicates. (**D**) qPCR of *Atf3*, *Atf4*, and *Atf5* expression in 10 µM and 100 µM serotonin treated BJ fibroblasts normalized to vehicle control. Multiple unpaired t-tests with Welch's correction showed no significant differences. N=3 biological replicates. (**E**) qPCR of *Asns*, *Chac1*, *Pck2*, *Psph*, *Hspa9*, and *Hspd1* expression in 10 µM and 100 µM serotonin treated BJ fibroblasts normalized to vehicle control. Multiple unpaired t-tests with Welch's correction showed no significant differences. N=3 biological replicates.

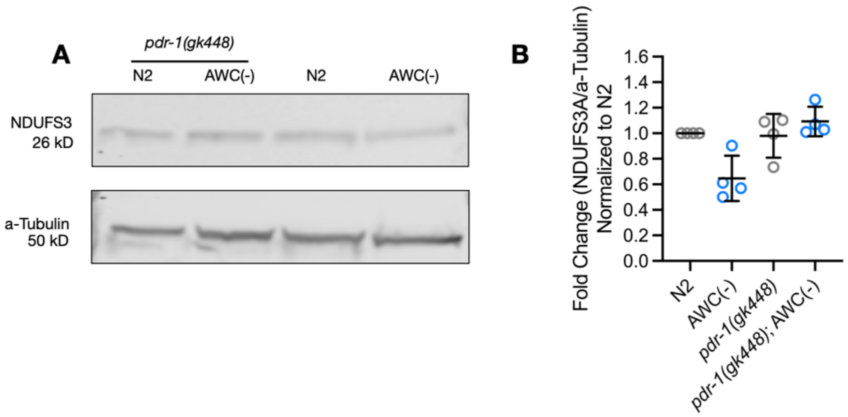


**Supplementary Figure 4. AWC(-) animals exhibit lower NDUFS3 protein levels.**
(**A**) Immunoblot analysis of *pdr-1(gk448)*, *pdr-1(gk448)*; AWC(-), N2, and AWC(-) at L4 stage. Blot shows anti-NDUFS3 (electron transport chain (ETC) complex I protein subunit) and anti-α-Tubulin. (**B)** Fold change of NDUFS3 fluorescence intensity / α-Tubulin fluorescence intensity in AWC(-), *pdr-1(gk448)*, and *pdr-1(gk448)*; AWC(-) normalized to N2 within experiments. AWC(-) average value = 0.647; *pdr-1(gk448)* average value = 0.9804; *pdr-1(gk448)*; AWC(-) average value = 1.093. N=4 biological replicates.

**Supplementary Table 1. Strain list.**

| Strain | Source | Identifier |
|---|---|---|
| *C. elegans*: strain N2 (Bristol) | CGC (Caenorhabditis Genetics Center) | N2 |
| *C. elegans*: strain PY7502, *oyIs85 [ceh-36p::TU#813 + ceh-36p::TU#814 + srtx-1p::GFP + unc-122p::DsRed].* | CGC | AWC(-) |
| *C. elegans*: strain AGD3126, *zcIs13 [hsp-6p::GFP] V.* | CGC and the Dillin Laboratory | *hsp-6::GFP* |
| *C. elegans*: strain AGD3644, *oyIs85 [ceh-36p::TU#813 + ceh-36p::TU#814 + srtx-1p::GFP + unc-122p::DsRed]; zcIs13[hsp-6p::GFP].* | This paper | AWC(-); *hsp-6::GFP* |
| *C. elegans*: strain AGD1960, *atfs-1(gk3094) V.* | CGC and the Dillin Laboratory | *atfs-1(gk3094)* |
| *C. elegans*: strain AGD3695, *atfs-1(gk3094) V; oyIs85 [ceh-36p::TU#813 + ceh-36p::TU#814 + srtx-1p::GFP + unc-122p::DsRed]* | This paper | *atfs-1(gk3094);* AWC(-) |
| *C. elegans*: strain SJZ204, *foxSi37 [ges-1p::tomm-20::mKate2::HA::tbb-2 3' UTR] I.* | CGC | *ges-1p::tomm-20::mKate2* |
| *C. elegans*: strain AGD3782, *foxSi37 [ges-1p::tomm-20::mKate2::HA::tbb-2 3' UTR] I.; oyIs85 [ceh-36p::TU#813 + ceh-36p::TU#814 + srtx-1p::GFP + unc-122p::DsRed]* | This paper | AWC(-); *ges-1p::tomm-20::mKate2* |
| *C. elegans*: strain AGD3697, *Ex[pceh-36::HisCl; pmyo-3::gfp]*. Backcrossed to N2 3x to remove *hhIs64 [unc-119(+); sur-5::UbiV-GFP]III.* | This paper and gift from the Hoppe Laboratory (*9*) | *ceh-36p::HisCl* |
| *C. elegans*: strain AGD3871, *Ex[pceh-36::HisCl; pmyo-3::gfp]; zcIs13[hsp-6p::GFP] V* | This paper | *ceh-36p::HisCl; hsp-6::GFP* |
| *C. elegans*: strain EG9631, *unc-13(s69) I.* | CGC | *unc-13(s69)* |

| | | |
|---|---|---|
| *C. elegans*: strain AGD3415, *unc-13(s69); zcIs13[hsp-6::GFP]* | The Dillin Laboratory | *unc-13(s69); hsp-6::GFP* |
| *C. elegans*: strain AGD3872, *unc-13(s69) I; oyIs85 [ceh-36p::TU#813 + ceh-36p::TU#814 + srtx-1p::GFP + unc-122p::DsRed]* | This paper | *unc-13(s69);* AWC(-) |
| *C. elegans*: strain AGD3873, *unc-13(s69) I; oyIs85 [ceh-36p::TU#813 + ceh-36p::TU#814 + srtx-1p::GFP + unc-122p::DsRed]; zcIs13[hsp-6::GFP]* | This paper | *unc-13(s69);* AWC(-); *hsp-6::GFP* |
| *C. elegans*: strain AG2225, *unc-31(e928) IV.* | The CGC and Dillin Laboratory | *unc-31(e928)* |
| *C. elegans*: strain AGD3002, *unc-31(e928) IV; ); zcIs13[hsp-6::GFP].* | The Dillin Laboratory | *unc-31(e928); hsp-6::GFP* |
| *C. elegans*: strain AGD3630, *unc-31(e928)IV; oyIs85 [ceh-36p::TU#813 + ceh-36p::TU#814 + srtx-1p::GFP + unc-122p::DsRed]* | This paper | *unc-31(e928);* AWC(-) |
| *C. elegans*: strain AGD3641, *unc-31(e928)IV; oyIs85 [ceh-36p::TU#813 + ceh-36p::TU#814 + srtx-1p::GFP + unc-122p::DsRed]; zcIs13[hsp-6p::GFP]* | This paper | *unc-31(e928)*, AWC(-); *hsp-6::GFP* |
| *C. elegans*: strain AGD3039, *tph-1(mg280) II.* | The CGC and Dillin Laboratory | *tph-1(mg280)* |
| *C. elegans*: strain AGD3466, *zcIs13[hsp-6p::GFP] V; tph-1(mg280) II.* | The Dillin Laboratory | *tph-1(mg280); hsp-6::GFP* |
| *C. elegans*: strain AGD3874, *tph-1(mg280) II; oyIs85 [ceh-36p::TU#813 + ceh-36p::TU#814 + srtx-1p::GFP + unc-122p::DsRed]* | This paper | *tph-1(mg280);* AWC(-) |
| *C. elegans*: strain AGD3875, *tph-1(mg280) II; oyIs85 [ceh-36p::TU#813 + ceh-36p::TU#814 + srtx-1p::GFP + unc-122p::DsRed]; zcIs13[hsp-6::GFP]* | This paper | *tph-1(mg280);* AWC(-); *hsp-6::GFP* |
| *C. elegans*: strain VC1024, *pdr-1(gk448) III.* | The CGC | *pdr-1(gk448)* |

| | | |
|---|---|---|
| *C. elegans*: strain AGD3632, *pdr-1(gk448) III; oyIs85 [ceh-36p::TU#813 + ceh-36p::TU#814 + srtx-1p::GFP + unc-122p::DsRed]* | This paper | *pdr-1(gk448);* AWC(-) |
| *C. elegans*: strain AGD3399, *pdr-1(gk448) III; zcIs13[hsp-6p::GFP]* | The Dillin Laboratory | *pdr-1(gk448); hsp-6::GFP* |
| *C. elegans:* strain AGD3648, *pdr-1(gk448); hsp-6p::GFP; oyIs85 [ceh-36p::TU#813 + ceh-36p::TU#814 + srtx-1p::GFP + unc-122p::DsRed]* | This paper | *pdr-1(gk448);* AWC(-); *hsp-6::GFP* |

**Supplementary Table 2. qPCR target primers.**

| Target gene | Forward sequence | Reverse sequence |
|---|---|---|
| ATF3 | CCTCTGCGCTGGAATCAGTC | TTCTTTCTCGTCGCCTCTTTTT |
| ATF4 | CTCCGGGACAGATTGGATGTT | GGCTGCTTATTAGTCTCCTGGAC |
| ATF5 | TGGCTCGTAGACTATGGGAAA | ATCAACTCGCTCAGTCATCCA |
| ASNS | GGAAGACAGCCCCGATTTACT | AGCACGAACTGTTGTAATGTCA |
| PCK2 | GCCATCATGCCGTAGCATC | AGCCTCAGTTCCATCACAGAT |
| PSPH | GAGGACGCGGTGTCAGAAAT | GGTTGCTCTGCTATGAGTCTCT |
| HSPA9 | CTTGTTTCAAGGCGGGATTATGC | GCAGGAGTTGGTAGTACCCAAA |
| HSPD1 | CTACTGTACTGGCACGCTCTA | CAACAGCTAACATCACACCTCTC |
